# Supplementary material for: Wildlife Management Practices Associated with Pathogen Exposure in Non-Native Wild Pigs in Florida, U.S
Source: Viruses. 2018 Dec 26;11(1):14. doi: 10.3390/v11010014 (PMC6356989; doi:10.3390/v11010014)
Supplement: Supplementary file 1 [file viruses-11-00014-s001.pdf]

Table S1. Site-level pseudorabies virus virus (PrV) antibody detection probability ($\hat{p}$), corrected probability of occurrence (Psi_{cond}), sample size, and apparent seroprevalence for 15 wild pig sites with dog hunting and 68 sites with no hunting, 2007-2014 (Florida, U.S.A.).

Site	Policy	Year								$\hat{p}$	Psi_{cond}	Sample size	Apparent seroprevalence
		2007	2008	2009	2010	2011	2012	2013	2014				
1	Hunt	.	.	1	1	1	1	.	1	0.96	1.00	89	0.70
2	Hunt	.	.	.	.	.	.	.	1	0.96	1.00	1	1.00
3	Hunt	.	.	.	.	0	.	0	.	0.96	0.02	6	0.00
4	Hunt	.	.	.	.	.	1	1	.	0.96	1.00	19	0.68
5	Hunt	.	.	.	.	.	1	.	.	0.96	1.00	1	1.00
6	Hunt	1	1	1	1	1	1	1	1	0.96	1.00	297	0.29
7	Hunt	.	.	.	.	1	.	.	1	0.96	1.00	36	0.44
8	Hunt	.	.	1	1	1	1	1	1	0.96	1.00	116	0.53
9	Hunt	.	.	.	1	0	1	1	1	0.96	1.00	23	0.48
10	Hunt	.	1	.	1	.	.	.	1	0.96	1.00	21	0.48
11	Hunt	.	.	.	.	.	.	.	1	0.96	1.00	29	0.45
12	Hunt	.	.	.	1	1	1	1	1	0.96	1.00	102	0.57
13	Hunt	.	.	1	1	1	1	.	1	0.96	1.00	86	0.52
14	Hunt	.	.	.	.	0	1	1	1	0.96	1.00	41	0.66
15	Hunt	.	.	.	1	1	1	1	1	0.96	1.00	28	0.79
16	No	.	.	.	.	.	.	1	1	0.72	1.00	18	0.22
17	No	.	0	.	.	.	.	.	.	0.72	0.46	2	0.00
18	No	.	.	.	.	.	1	1	1	0.72	1.00	48	0.69
19	No	.	1	1	.	.	1	.	.	0.72	1.00	51	0.24
20	No	0	1	.	.	.	.	.	.	0.72	1.00	26	0.04
21	No	.	.	.	.	.	0	1	.	0.72	1.00	15	0.33
22	No	.	.	.	.	1	.	.	.	0.72	1.00	14	0.07
23	No	.	0	.	.	.	.	.	.	0.72	0.46	4	0.00
24	No	.	.	.	.	1	0	.	.	0.72	1.00	10	0.10
25	No	.	1	1	1	.	.	0	.	0.72	1.00	80	0.34
26	No	1	1	1	1	1	.	.	1	0.72	1.00	48	0.54

27	No	.	.	.	.	0	0	.	.	0.72	0.19	12	0.00
28	No	.	.	1	1	.	.	.	.	0.72	1.00	9	0.78
29	No	.	1	.	1	1	.	.	.	0.72	1.00	42	0.31
30	No	1	0	.	.	1	.	.	.	0.72	1.00	33	0.45
31	No	.	.	0	.	.	0	.	0	0.72	0.06	33	0.00
32	No	.	.	.	.	.	1	1	1	0.72	1.00	21	0.67
33	No	0	.	.	.	.	.	.	.	0.72	0.46	1	0.00
34	No	.	.	.	.	.	.	0	.	0.72	0.46	5	0.00
35	No	.	.	.	.	.	0	.	.	0.72	0.46	5	0.00
36	No	.	0	.	.	.	.	.	.	0.72	0.46	4	0.00
37	No	.	.	1	.	.	.	.	.	0.72	1.00	7	0.29
38	No	.	.	.	.	.	.	0	.	0.72	0.46	3	0.00
39	No	0	0	.	.	.	.	.	.	0.72	0.19	6	0.00
40	No	.	.	.	.	.	.	1	0	0.72	1.00	9	0.11
41	No	0	0	.	.	.	.	.	.	0.72	0.19	11	0.00
42	No	.	.	.	.	.	.	.	0	0.72	0.46	1	0.00
43	No	0	0	.	.	.	.	.	1	0.72	1.00	13	0.15
44	No	.	.	.	.	.	.	1	.	0.72	1.00	1	1.00
45	No	.	.	.	0	1	.	.	.	0.72	1.00	23	0.09
46	No	.	.	.	.	0	.	.	.	0.72	0.46	1	0.00
47	No	.	.	.	.	.	.	.	0	0.72	0.46	3	0.00
48	No	.	.	.	0	.	0	.	0	0.72	0.06	20	0.00
49	No	.	.	.	.	0	.	.	.	0.72	0.46	6	0.00
50	No	.	.	.	.	.	1	.	.	0.72	1.00	3	0.33
51	No	.	0	.	.	.	.	.	.	0.72	0.46	1	0.00
52	No	.	.	.	.	0	.	.	.	0.72	0.46	33	0.00
53	No	.	.	.	.	.	.	.	0	0.72	0.46	10	0.00
54	No	1	.	0	.	.	.	.	.	0.72	1.00	8	0.25
55	No	1	0	.	1	1	.	1	1	0.72	1.00	84	0.54
56	No	1	.	.	.	.	.	.	.	0.72	1.00	5	0.40
57	No	.	.	1	1	1	.	.	.	0.72	1.00	58	0.71
58	No	.	.	.	.	.	.	.	1	0.72	1.00	13	0.38

59	No	.	.	.	.	.	.	.	1	0.72	1.00	6	0.33
60	No	.	.	.	0	.	.	.	.	0.72	0.46	3	0.00
61	No	.	.	.	.	0	.	.	.	0.72	0.46	8	0.00
62	No	1	.	.	1	1	.	1	.	0.72	1.00	42	0.67
63	No	.	1	.	.	1	.	.	.	0.72	1.00	12	0.25
64	No	0	.	.	.	.	.	.	.	0.72	0.46	2	0.00
65	No	0	.	.	.	.	.	.	.	0.72	0.46	1	0.00
66	No	.	.	.	.	.	1	0	.	0.72	1.00	16	0.13
67	No	.	.	.	.	1	.	.	.	0.72	1.00	1	1.00
68	No	0	.	1	.	.	.	.	.	0.72	1.00	2	0.50
69	No	.	0	.	.	.	.	.	.	0.72	0.46	22	0.00
70	No	.	.	.	0	0	.	.	.	0.72	0.19	6	0.00
71	No	1	.	1	1	1	1	.	.	0.72	1.00	57	0.28
72	No	.	.	0	1	0	.	.	.	0.72	1.00	10	0.10
73	No	.	.	0	.	.	.	.	.	0.72	0.46	19	0.00
74	No	.	.	1	.	.	.	.	.	0.72	1.00	4	0.25
75	No	1	.	.	.	.	.	.	.	0.72	1.00	1	1.00
76	No	1	1	.	.	.	.	1	.	0.72	1.00	16	0.75
77	No	.	.	.	.	1	1	1	.	0.72	1.00	10	0.80
78	No	.	.	.	.	.	.	1	.	0.72	1.00	1	1.00
79	No	0	.	.	.	.	.	.	.	0.72	0.46	10	0.00
80	No	.	0	.	.	.	.	1	0	0.72	1.00	21	0.05
81	No	.	1	.	.	.	.	.	.	0.72	1.00	9	0.22
82	No	.	.	.	.	.	.	0	.	0.72	0.46	2	0.00
83	No	.	1	0	.	.	.	.	.	0.72	1.00	15	0.40
Hunt total										0.96	0.93	895	0.48
No hunt total										0.72	0.75	1096	0.32
Grand total										0.81	0.75	1991	0.39

Table S2. Site-level *Brucella* spp. antibody detection probability ($\hat{p}$), corrected probability of occurrence (Psi_{cond}), sample size, and apparent seroprevalence for 15 wild pig sites with dog hunting and 68 sites with no hunting, 2007-2014 (Florida, U.S.A.).

Site	Policy	Year								$\hat{p}$	Psi_{cond}	Sample size	Apparent seroprevalence
		2007	2008	2009	2010	2011	2012	2013	2014				
1	Hunt	.	.	1	1	0	1	.	1	0.73	1.00	89	0.18
2	Hunt	.	.	.	.	.	.	.	0	0.73	0.63	1	0.00
3	Hunt	.	.	.	.	0	.	0	.	0.73	0.32	6	0.00
4	Hunt	.	.	.	.	.	1	1	.	0.73	1.00	19	0.37
5	Hunt	.	.	.	.	.	1	.	.	0.73	1.00	1	1.00
6	Hunt	0	0	0	0	0	0	0	0	0.73	0.00	297	0.00
7	Hunt	.	.	.	.	1	.	.	1	0.73	1.00	36	0.31
8	Hunt	.	.	1	1	1	1	1	1	0.73	1.00	116	0.24
9	Hunt	.	.	.	0	0	1	0	1	0.73	1.00	23	0.26
10	Hunt	.	0	.	1	.	.	.	0	0.73	1.00	21	0.05
11	Hunt	.	.	.	.	.	.	.	1	0.73	1.00	29	0.34
12	Hunt	.	.	.	0	1	0	1	1	0.73	1.00	102	0.25
13	Hunt	.	.	1	0	1	1	.	1	0.73	1.00	86	0.17
14	Hunt	.	.	.	.	0	1	1	1	0.73	1.00	41	0.29
15	Hunt	.	.	.	0	1	1	1	1	0.73	1.00	28	0.36
16	No	.	.	.	.	.	.	1	1	0.56	1.00	18	0.33
17	No	.	0	.	.	.	.	.	.	0.56	0.38	2	0.00
18	No	.	.	.	.	.	1	1	1	0.56	1.00	48	0.15
19	No	.	0	0	.	.	0	.	.	0.56	0.10	51	0.00
20	No	0	1	.	.	.	.	.	.	0.56	1.00	26	0.15
21	No	.	.	.	.	.	1	0	.	0.56	1.00	15	0.13
22	No	.	.	.	.	0	.	.	.	0.56	0.38	14	0.00
23	No	.	0	.	.	.	.	.	.	0.56	0.38	4	0.00
24	No	.	.	.	.	0	0	.	.	0.56	0.21	10	0.00
25	No	.	1	1	1	.	.	1	.	0.56	1.00	80	0.13
26	No	1	1	1	0	1	.	.	0	0.56	1.00	48	0.25
27	No	.	.	.	.	0	0	.	.	0.56	0.21	12	0.00

28	No	.	.	1	0	.	.	.	.	0.56	1.00	9	0.22
29	No	.	0	.	0	0	.	.	.	0.56	0.10	42	0.00
30	No	0	0	.	.	0	.	.	.	0.56	0.10	33	0.00
31	No	.	.	0	.	.	0	.	0	0.56	0.10	33	0.00
32	No	.	.	.	.	.	0	1	0	0.56	1.00	21	0.19
33	No	0	.	.	.	.	.	.	.	0.56	0.38	1	0.00
34	No	.	.	.	.	.	.	0	.	0.56	0.38	5	0.00
35	No	.	.	.	.	.	0	.	.	0.56	0.38	5	0.00
36	No	.	0	.	.	.	.	.	.	0.56	0.38	4	0.00
37	No	.	.	1	.	.	.	.	.	0.56	1.00	7	0.29
38	No	.	.	.	.	.	.	1	.	0.56	1.00	3	0.67
39	No	0	0	.	.	.	.	.	.	0.56	0.21	6	0.00
40	No	.	.	.	.	.	.	0	1	0.56	1.00	9	0.22
41	No	1	0	.	.	.	.	.	.	0.56	1.00	11	0.09
42	No	.	.	.	.	.	.	.	0	0.56	0.38	1	0.00
43	No	1	1	.	.	.	.	.	0	0.56	1.00	13	0.15
44	No	.	.	.	.	.	.	1	.	0.56	1.00	1	1.00
45	No	.	.	.	0	0	.	.	.	0.56	0.21	23	0.00
46	No	.	.	.	.	0	.	.	.	0.56	0.38	1	0.00
47	No	.	.	.	.	.	.	.	0	0.56	0.38	3	0.00
48	No	.	.	.	1	.	0	.	0	0.56	1.00	20	0.05
49	No	.	.	.	.	1	.	.	.	0.56	1.00	6	0.17
50	No	.	.	.	.	.	1	.	.	0.56	1.00	3	0.67
51	No	.	0	.	.	.	.	.	.	0.56	0.38	1	0.00
52	No	.	.	.	.	0	.	.	.	0.56	0.38	33	0.00
53	No	.	.	.	.	.	.	.	0	0.56	0.38	10	0.00
54	No	1	.	0	.	.	.	.	.	0.56	1.00	8	0.13
55	No	1	0	.	1	0	.	1	1	0.56	1.00	84	0.23
56	No	1	.	.	.	.	.	.	.	0.56	1.00	5	0.20
57	No	.	.	1	1	0	.	.	.	0.56	1.00	58	0.10
58	No	.	.	.	.	.	.	.	1	0.56	1.00	13	0.31
59	No	.	.	.	.	.	.	.	0	0.56	0.38	6	0.00

60	No	.	.	.	0	.	.	.	.	0.56	0.38	3	0.00
61	No	.	.	.	.	0	.	.	.	0.56	0.38	8	0.00
62	No	0	.	.	0	1	.	1	.	0.56	1.00	42	0.14
63	No	.	1	.	.	0	.	.	.	0.56	1.00	12	0.08
64	No	1	.	.	.	.	.	.	.	0.56	1.00	2	0.50
65	No	0	.	.	.	.	.	.	.	0.56	0.38	1	0.00
66	No	.	.	.	.	.	1	1	.	0.56	1.00	16	0.31
67	No	.	.	.	.	0	.	.	.	0.56	0.38	1	0.00
68	No	0	.	0	.	.	.	.	.	0.56	0.21	2	0.00
69	No	.	0	.	.	.	.	.	.	0.56	0.38	22	0.00
70	No	.	.	.	0	0	.	.	.	0.56	0.21	6	0.00
71	No	0	.	0	0	0	0	.	.	0.56	0.02	57	0.00
72	No	.	.	0	0	0	.	.	.	0.56	0.10	10	0.00
73	No	.	.	0	.	.	.	.	.	0.56	0.38	19	0.00
74	No	.	.	0	.	.	.	.	.	0.56	0.38	4	0.00
75	No	0	.	.	.	.	.	.	.	0.56	0.38	1	0.00
76	No	1	0	.	.	.	.	1	.	0.56	1.00	16	0.25
77	No	.	.	.	.	0	0	0	.	0.56	0.10	10	0.00
78	No	.	.	.	.	.	.	0	.	0.56	0.38	1	0.00
79	No	1	.	.	.	.	.	.	.	0.56	1.00	10	0.20
80	No	.	0	.	.	.	.	0	0	0.56	0.10	21	0.00
81	No	.	0	.	.	.	.	.	.	0.56	0.38	9	0.00
82	No	.	.	.	.	.	.	0	.	0.56	0.38	2	0.00
83	No	.	0	0	.	.	.	.	.	0.56	0.21	15	0.00
Hunt total										0.73	0.86	895	0.16
No hunt total										0.56	0.58	1096	0.10
Grand total										0.63	0.63	1991	0.13
